# Intermittent Theta Burst Stimulation Improves Sleep in Autism Spectrum Disorder by Reorganizing Overlapping Brain Networks With Neurochemical and Transcriptomic Signatures: A Randomized Controlled Trial

**DOI:** 10.1002/cns.71041

**Published:** 2026-08-12

**Authors:** Huashuang Zhang, Bincan Xiong, Hongchi Liu, Yilin Huang, Jingwen Zhang, Ruimin Yu, Wenqi Jiang, Tongjia She

**Affiliations:** ^1^ The Third Affiliated Hospital, School of Medicine Foshan University Foshan Guangdong China; ^2^ Institute for Brain Research and Rehabilitation South China Normal University Guangzhou Guangdong China

**Keywords:** autism spectrum disorder, biological context, insomnia, theta burst stimulation

## Abstract

**Background:**

Overlapping network architecture supports functional integration and multifunctional regional engagement in the brain, and may serve as a candidate biomarker for interventions in autism spectrum disorder (ASD). However, the biological context underlying intermittent theta‐burst stimulation (iTBS)‐related changes in overlapping network architecture remains poorly understood.

**Methods:**

Seventy patients with ASD and chronic insomnia were randomly assigned to receive either real or sham iTBS targeting the left orbitofrontal cortex, administered once daily for 8 weeks. The Insomnia Severity Index (ISI) and resting‐state functional magnetic resonance imaging (fMRI) data were collected at baseline and after the intervention. The Shannon–entropy diversity coefficient was calculated to characterize overlapping network architecture. The JuSpace toolbox was used to assess spatial correspondence between iTBS‐related network changes and neurotransmitter systems. Transcriptomic–neuroimaging association analyses were then performed using gene‐expression data from the Allen Human Brain Atlas.

**Results:**

Sleep improvement following real iTBS was accompanied by changes in overlapping network architecture across 13 cortical regions. These changes showed spatial correspondence with mGluR5 and GABA_A receptor density maps. Regions showing iTBS‐related network changes were associated with glutamatergic synaptic transmission and calcium‐dependent signaling, enriched for cortical excitatory neuronal signatures with peak expression during adolescence, and organized into a highly interconnected protein–protein interaction network centered on the Ca^2+^–PKA signaling axis.

**Conclusions:**

This study provides multiscale evidence for the biological context underlying iTBS‐related changes in overlapping network architecture in ASD with chronic insomnia.

## Graphical abstract

iTBS improves insomnia in ASD by reorganizing the brain's overlapping network architecture. These changes align with mGluR5/GABAA receptor distributions and are driven by a gene‐regulatory network centered on the Ca^2+^–PKA signaling axis, highlighting a developmentally calibrated synaptic tuning mechanism.

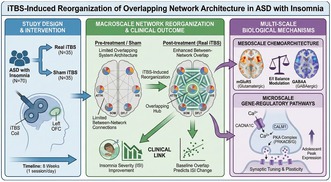

## 1 Introduction

Autism spectrum disorder (ASD) remains a major challenge for therapeutic development due to its complex neurobiological underpinnings and heterogeneous clinical presentation. Increasing evidence indicates that chronic insomnia is not merely a comorbid condition but is integral to the core pathophysiology of ASD, contributing to neural dysregulation across multiple systems and directly exacerbating core ASD symptoms, including severe irritability, repetitive behaviors, and impaired social communication [1, 2, 3]. Addressing these sleep‐related abnormalities is therefore essential for improving clinical outcomes and advancing mechanistic understanding of ASD. Intermittent theta‐burst stimulation (iTBS), a non‐invasive neuromodulatory technique capable of inducing long‐term potentiation (LTP)‐like synaptic plasticity, offers a promising avenue to restore the excitation‐inhibition balance and normalize network communication [4, 5]. We hypothesized that focal iTBS may be associated with system‐level alterations in large‐scale network organization, potentially reflected by changes in network diversity. Understanding the brain network mechanisms mediating iTBS‐induced modulation represents a crucial step toward optimizing its therapeutic efficacy for ASD.

Growing neuroimaging evidence conceptualizes ASD as a disconnection syndrome involving disrupted interactions among large‐scale brain systems [6, 7, 8]. However, most earlier connectome studies employed non‐overlapping network models that assume each brain region belongs to a single discrete network, thereby overlooking spatially overlapping organization [9]. In contrast, evidence indicates that many brain regions simultaneously contribute to multiple subnetworks, forming an overlapping topology supporting cross‐system communication [9]. Such regions typically display cross‐network coactivation, hub‐like structural properties, and high dynamic adaptability, while disruption of these overlapping hubs has been linked to impaired large‐scale integration in neuropsychiatric conditions [10, 11]. To capture this aspect of system‐level organization, we modeled the brain's overlapping architecture using the Shannon‐entropy diversity coefficient (H), an information‐theoretic index that quantifies how evenly a node's connectivity is distributed across functional modules [12]. Higher H indicates broader cross‐system participation, whereas lower H reflects more module‐specific connectivity [12]. Because iTBS‐induced plasticity may involve not only changes in local coupling strength but also redistribution of regional participation across overlapping cortical systems, the H provides a theoretically appropriate measure for examining stimulation‐related network reorganization.

Integrating neuroimaging data with neurotransmitter systems offers a powerful framework for elucidating the biological mechanisms of brain disorders [13, 14, 15]. Evidence increasingly shows functional heterogeneity in the human brain is constrained by its intrinsic chemoarchitectural scaffold [16]. An imbalance between excitation and inhibition (E/I) has been recognized as a shared pathophysiological mechanism underlying both ASD and insomnia [17]. This imbalance is shaped by regional variations in glutamatergic (mGluR5) and GABAergic (GABA_A) receptor densities, which modulate local E/I balance and consequently large‐scale network connectivity [18]. Our previous work demonstrated that brain stimulation can alleviate insomnia symptoms by regulating GABAergic neurotransmission [19], underscoring the therapeutic relevance of targeting the E/I system. The JuSpace toolbox [20], which incorporates normative positron emission tomography (PET)‐derived neurotransmitter maps, enables testing whether ASD‐related network alterations align with underlying neurotransmitter architectures, thereby facilitating cross‐scale analyses of functional‐neurochemical correspondence [21]. Integrating metrics of network complexity (e.g., Shannon‐entropy diversity) with neurotransmitter profiles can offer new insights into neurobiological mechanisms within a trans‐diagnostic framework.

Additionally, transcriptome‐connectome association studies have established a framework linking macroscale brain networks with microscale transcriptional profiles [22]. This framework has subsequently been applied to a range of neurological and psychiatric conditions, such as major depressive disorder [23] and Parkinson's disease [24], offering important insights into how gene expression gradients influence disease‐related network reorganization. However, no study has yet investigated these transcriptome‐connectome associations in ASD with chronic insomnia. The biological context underlying the alterations in the overlapping system‐level architecture remains largely unknown.

Therefore, we conducted a randomized, double‐blind, sham‐controlled trial involving 70 ASD with chronic insomnia to investigate the neurobiological mechanisms underlying the therapeutic effects of iTBS. Specifically, we aimed to test three hypotheses: (1) iTBS induces reorganization of the brain's overlapping system‐level architecture, which exhibits regional specificity across large‐scale functional networks and is associated with improvements in insomnia symptoms in ASD. (2) The spatial pattern of iTBS‐induced network reorganization corresponds to regional densities of glutamatergic (mGluR5) and GABAergic (GABA_A) receptors, reflecting modulation of the E/I balance. (3) The spatial distribution of iTBS‐related alterations in network integration is correlated with the receptor topography and expression maps of specific genes, providing molecular insights into the multiscale mechanisms underlying iTBS modulation in ASD with chronic insomnia.

## 2 Methods

### 2.1 Study Design

**FIGURE 1:**
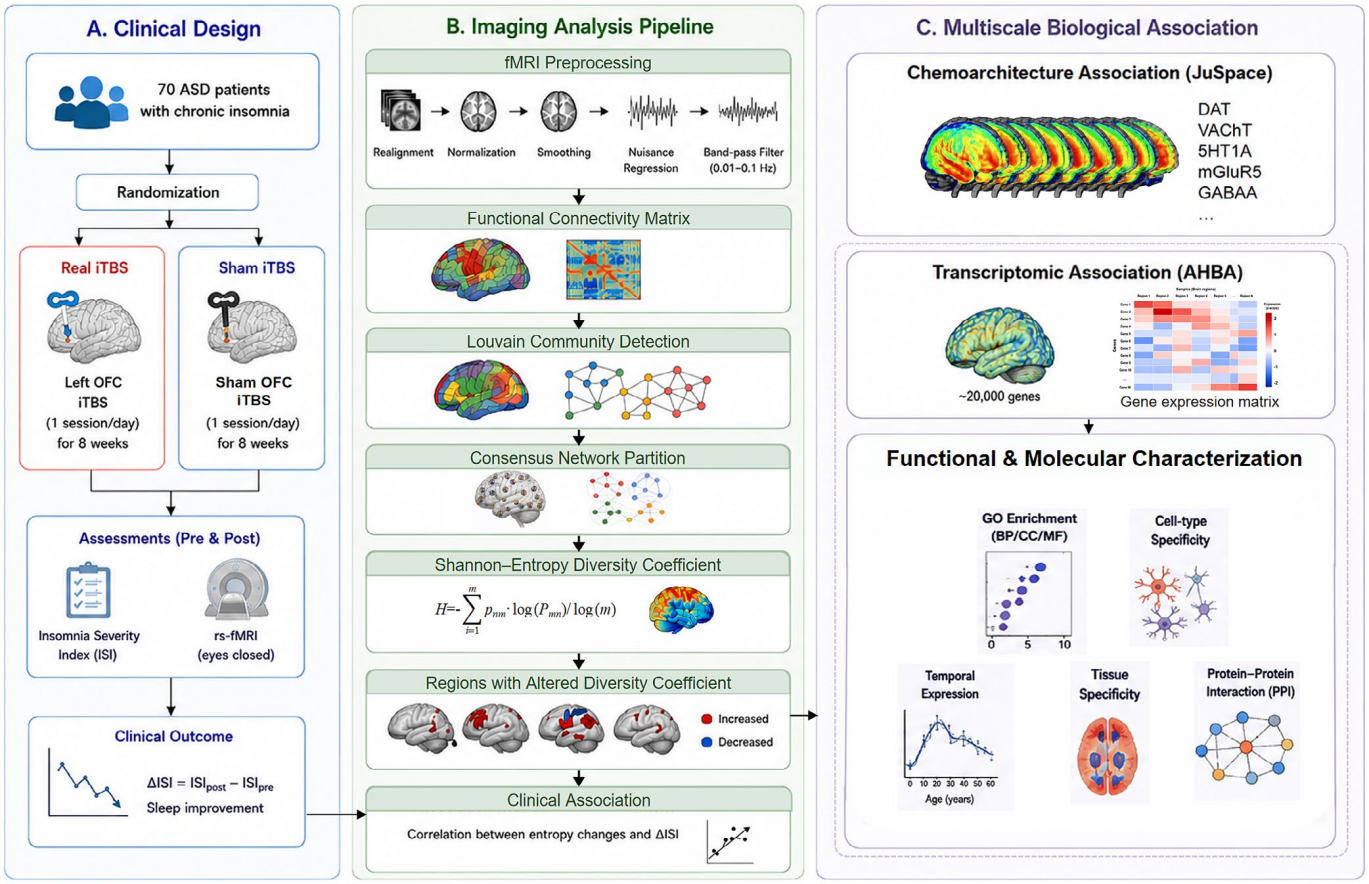
Overview of the analysis pipeline. ASD, autism spectrum disorder; iTBS, intermittent theta burst stimulation; OFC, orbitofrontal cortex; rs‐fMRI, resting‐state functional magnetic resonance imaging; ISI, Insomnia Severity Index; ΔISI, changes in ISI; H, Shannon‐entropy diversity coefficient; DAT, dopamine transporter; VAChT, vesicular acetylcholine transporter; 5‐HT1A, serotonin 1A receptor; mGluR5, metabotropic glutamate receptor 5; GABA_A, gamma‐aminobutyric acid type A receptor; AHBA, Allen Human Brain Atlas; GO, Gene Ontology; BP, biological process; CC, cellular component; MF, molecular function; PPI, protein–protein interaction.

This research implemented an 8‐week randomized, double‐blind, controlled design involving two parallel groups: (A) real iTBS and (B) sham iTBS. For all outcomes, assessments were conducted at baseline and repeated after the completion of the 8‐week course of intervention. Specifically, the Insomnia Severity Index (ISI) and functional magnetic resonance imaging (fMRI) were administered at baseline and post‐intervention. The stimulation was delivered once daily, five consecutive days per week. The specific analytic framework is shown in Figure 1.

### 2.2 Participants

The recruitment of participants from the local community was conducted between March 2024 and July 2025, using advertisements, public lectures, and clinical referrals (Table 1). Eligibility screening was performed by physicians at the TPHS, China. Individuals who met the inclusion criteria were invited to an informational session, during which written informed consent was obtained. Inclusion criteria required: (1) an age range of 18–60 years; (2) a clinical diagnosis of ASD in accordance with DSM‐5 criteria [25]; (3) a DSM‐5 clinical diagnosis of chronic insomnia; (4) ISI scoring > 7 prior to therapy; (5) Dosage of hypnotics remained stable for the initial 4 treatment weeks; (6) right hand dominance; (7) signed a written informed consent form. The exclusion criteria were: (1) individuals with other untreated sleep disorders including obstructive sleep apnea or hypersomnia; (2) individuals who are pregnant or lactating; (3) currently or previously participated in other trials; (4) individuals with a history of seizures or serious head injury; (5) intellectual disability (IQ ≤ 70); (6) severe language impairment.

**TABLE 1:** Demographics and clinical characteristics.

Characteristics	Real stimulation (*n* = 35)	Sham stimulation (*n* = 35)	t/*x* ^2^	*p*
Age (years)	33.78 ± 11.22	34.14 ± 11.25	−0.13	0.890
Gender			1.56	0.212
Male	20	25		
Female	15	10		
FSIQ	116.93 ± 9.12	120.69 ± 14.23	−1.32	0.190
ADOS	10.80 ± 3.91	11.51 ± 4.13	−0.74	0.460
Insomnia duration (years)	9.25 ± 1.72	9.21 ± 1.20	0.11	0.910
Daily quantity of hypnotics used (diazepam equivalent, mg)	1.30 ± 2.48	1.56 ± 2.82	−0.41	0.680

Abbreviations: ADOS, Autism Diagnostic Observation Schedule; FSIQ, full‐scale intelligence quotient.

### 2.3 Sample Size

The required sample size was estimated in advance using G*Power 3.1 [26] for a 2 × 2 repeated‐measures ANOVA. Assuming a medium effect size according to Cohen's criteria (f = 0.25, corresponding to ŋ^2^≈0.06), a significance level of α = 0.05, 90% statistical power, two assessment timepoints, and an expected within‐subject correlation of 0.5, the analysis yielded a minimum total sample of 44 individuals (22 per arm). Allowing for an anticipated 20% dropout rate, the target enrollment was increased to 27 participants per group. To further safeguard the study's power in the event of greater attrition, we ultimately recruited 35 participants for each arm.

### 2.4 Randomization and Blinding

Eligible participants were assigned to either the real iTBS or sham iTBS group in a 1:1 ratio at random. Randomization was conducted using a computer‐generated sequence of random numbers prepared by a statistician blinded to the experimental procedures. Both participants and investigators responsible for assessment were kept blind to treatment allocation. The operator administering iTBS was necessarily unblinded due to the technical requirements of stimulation delivery. To preserve blinding integrity, participants were informed that they would receive iTBS but were not told whether it was real or sham stimulation. They were also instructed not to discuss treatment details with blinded assessors. Blinding efficacy was evaluated after treatment completion by asking participants to guess their group assignment. Accuracy did not exceed chance level (*p* > 0.100), confirming effective masking.

### 2.5 Intervention: iTBS

Resting motor threshold (RMT) was determined according to established procedures [27]. The figure‐of‐eight coil (YIRUIDE Company Limited) was positioned stereotaxically over a neuronavigation‐defined left orbitofrontal cortex (OFC) anatomical target (see Supplementary Methods for navigation details).

The iTBS protocols were adapted from established clinical conventions to enhance participant tolerability and compliance, as demonstrated in prior studies with clinical populations [28, 29, 30]. Each iTBS session commenced with a ramp‐up block (600 pulses), during which the stimulation intensity was gradually increased from 0% to the target amplitude, ensuring that only a minimal number of pulses were delivered at a fully efficacious level. The real stimulation block (600 pulses) was then administered at 110% of each participant's RMT, or at the highest amplitude tolerated during the ramp‐up procedure. Drawing on evidence from TBS clinical trials [31, 32] and electric‐field modeling, amplitudes of ≥ 85% RMT were considered sufficient to elicit meaningful neuromodulatory effects. Participants who were unable to complete the real iTBS block at or above this threshold were excluded from the final dataset.

Across all sessions, stimulation followed a standard theta‐burst configuration, consisting of triplet bursts at 50 Hz delivered at a 5 Hz repetition rate. The real iTBS protocol included 20 trains, each 2 s in duration with an 8 s inter‐train pause, producing a total stimulation time of 192 s per session. For the sham condition, the identical stimulation protocol was applied over the same target but at 20% of the maximum stimulator output. This corresponded to 32%–57% RMT, a level below the threshold for inducing reliable cortical effects, thereby ensuring comparable scalp sensation and auditory cues between real and sham conditions without producing significant neurophysiological changes. The total treatment cycle spanned 8 weeks, with participants receiving one session per day, 5 days per week, for a total of 40 sessions.

### 2.6 Outcome Measures

#### 2.6.1 Primary Outcome: fMRI

The Philips 3.0 T MRI scanner (Philips Medical Systems) was used to acquire all fMRI. And the parameters for imaging were as follows: repetition time (TR) = 2000 ms, echo time (TE) = 30 ms, slice thickness = 4.0 mm, flip angle = 90°, matrix size = 64 × 64, number of slices = 35, and spatial resolution = 3 × 3 × 3 mm^3^.

#### 2.6.2 fMRI Preprocessing

Resting‐state data were preprocessed using the GRETNA toolbox [33] running within the SPM12 environment. To reduce magnetization instability at the beginning of each scan, the initial volumes (first five) were discarded. Head movement was subsequently corrected by realigning all images with a rigid‐body algorithm. Participants showing substantial motion—operationalized as either translation > 3 mm or mean framewise displacement (FD) above 0.5 mm—were excluded, resulting in the removal of two individuals from the real iTBS arm and one from the sham arm. After realignment, the functional series underwent temporal filtering (0.01–0.08 Hz) and nuisance regression using a unified linear model. This model included the 24‐parameter motion expansion, along with time courses extracted from white‐matter and cerebrospinal‐fluid masks and the whole‐brain signal, following recommended procedures to reduce physiological and scanner‐related noise [34]. White‐matter and cerebrospinal fluid (CSF) masks were derived from SPM12 probabilistic tissue priors (threshold = 0.9), and the global signal was taken from the default whole‐brain template. Finally, functional images were spatially normalized to the Montreal Neurological Institute (MNI) template by applying the deformation fields estimated during each participant's structural segmentation, and the normalized images were resampled to 3‐mm isotropic voxels.

#### 2.6.3 Secondary Outcome: ISI

The ISI is a 7‐item self‐report measure commonly used to assess the severity of insomnia‐related symptoms across nighttime difficulties and daytime functioning [35]. Each item is rated on a 5‐point Likert scale from 0 (no difficulty) to 4 (very severe difficulty), yielding a total score ranging from 0 to 28. Standard interpretive ranges classify scores of 0–7 as no clinically significant insomnia, 8–14 as mild or subthreshold insomnia, 15–21 as moderate insomnia, and 22–28 as severe insomnia [36]. It is an effective and dependable intervention, notably characterized by its susceptibility to variations in insomnia [37, 38].

### 2.7 Shannon‐Entropy Diversity Coefficient Analysis

Following preprocessing, a functional connectivity (FC) representation was generated for each participant. Cortical time series were extracted based on the 200‐parcel Schaefer2020 parcellation, which organizes regions into 17 large‐scale functional systems. For each parcel, the mean BOLD signal was obtained, and connectivity estimates were derived by correlating the time courses of all parcel pairs using Pearson correlation. The resulting correlation values were subsequently transformed into z‐scores via Fisher's r‐to‐z conversion to stabilize variance and approximate normality.

To identify a robust modular structure for subsequent diversity analysis, we employed the Louvain algorithm (resolution parameter γ = 1) from the MATLAB‐based brain connectivity toolbox. This algorithm was applied 1000 times to each participant's FC matrix to account for the stochasticity inherent in the optimization process. To derive a stable consensus partition from these 1000 solutions, a co‐assignment matrix was generated, which recorded the frequency with which each pair of nodes was assigned to the same community across all iterations. A final, representative partition for each individual was then obtained by applying a consensus threshold of 0.5 to this co‐assignment matrix. Subsequently, group‐level consensus partitions were generated by aggregating the individual‐level partitions within the real iTBS group and sham iTBS group, respectively.

Finally, based on these stable group‐level partitions, the H was computed for each node. This coefficient, derived from information theory, quantifies the diversity of a node's connections across all detected communities, reflecting the extent to which a node participates in multiple overlapping functional systems. The formula for H is as follows:

$$H=-\sum_{i=1}^{m}p_{\mathrm{nm}}\cdot \log\left(P_{\mathrm{mn}}\right)/\log\left(m\right)$$

where $P_{\mathrm{nm}}$ represents the proportion of connection strength from node n to module m, and m is the total number of modules.

### 2.8 Statistical Analysis

All analyses were conducted in the intent‐to‐treat sample (*N* = 70) using the last observation carried forward method to account for missing data. Statistical analyses were performed using SPSS version 22.0 (IBM Corp). Continuous variables are presented as mean ± standard deviation. Normality was tested with the Kolmogorov–Smirnov test. Statistical significance was set at a two‐sided *p* < 0.05.

*Demographic and clinical variables*. Baseline demographic and clinical characteristics were compared between the real and sham iTBS groups using two‐sample *t*‐tests, chi‐square tests, or Mann–Whitney U tests depending on data type and distribution.

*Clinical outcome analysis*. Changes in ISI scores from baseline to post‐treatment were analyzed using a linear mixed‐effects model. ISI score was the dependent variable. Fixed effects included group, time, and the group × time interaction, with participant specified as a random effect. The group × time interaction was the main effect of interest. Age, sex, baseline ISI score, and diazepam‐equivalent hypnotic dose were added as clinically relevant baseline covariates.

*Neuroimaging analysis*. To detect iTBS‐related changes in overlapping system‐level integration, H values were analyzed for each cortical parcel at baseline and post‐treatment in both groups. For descriptive purposes, within‐subject changes in H were calculated separately for the real and sham groups as follows:

$$\begin{array}{c} \Delta H_{\text{real}}=H_{\text{post},\text{real}}-H_{\mathrm{pre},\text{real}} \end{array}$$

$$\begin{array}{c} \Delta H_{\text{sham}}=H_{\text{post},\text{sham}}-H_{\mathrm{pre},\text{sham}} \end{array}$$

The difference in mean change between groups was used as a descriptive index of treatment‐related alterations in overlapping network:

$$\Delta H_{\text{iTBS}}={\bar{\mathrm{\Delta H}}}_{\text{real}}-{\bar{\mathrm{\Delta H}}}_{\text{sham}}$$

Formal statistical testing was performed using a 2 × 2 repeated‐measures ANOVA. Group (real vs. sham iTBS) was entered as the between‐subject factor, and time (baseline vs. post‐treatment) was entered as the within‐subject factor. The group × time interaction was the main effect of interest. FDR correction was applied across cortical parcels. FDR correction was applied across cortical parcels, and parcels surviving an FDR‐corrected threshold of *p* < 0.05 were defined as differential overlapping regions.

*Network‐clinical association*. Within the real iTBS group, partial correlations were used to test the associations between regional ΔH and ΔISI. ΔH and ΔISI were defined as post‐treatment minus baseline H and ISI score, respectively. Each model adjusted for age, sex, baseline ISI score, baseline H of the corresponding region, and daily diazepam‐equivalent hypnotic dose. Two‐tailed *p* values were reported, and FDR correction was applied across regions. Results with FDR‐adjusted *p* < 0.05 were considered significant.

*Support vector regression (SVR) analysis*. An exploratory SVR analysis was performed in the real iTBS group using LIBSVM [39]. We tested whether baseline H values from sleep improvement‐related regions predicted subsequent ΔISI. Baseline regional H values were entered as features, and ΔISI was used as the target variable. Baseline ISI score was included as a covariate. Prediction performance was evaluated with leave‐one‐out cross‐validation and quantified using the Pearson correlation between predicted and observed ΔISI. Statistical significance was tested by permutation.

### 2.9 Spatial Concordance With Neurotransmitter Receptor Maps

We assessed the spatial correspondence between iTBS‐related functional reorganization in ASD patients with insomnia and the spatial distributions of six neurotransmitter receptor or transporter systems using JuSpace (v1.5) [20]. Multiple comparisons were controlled using FDR adjustment. Density maps for these molecular systems, including D2R [40], DAT [41], mGluR5 [16], VAChT [16], 5‐HT1A [42] and GABA_A [18] were derived from positron‐emission tomography imaging.

### 2.10 Transcription–Neuroimaging Association Analysis

*Gene expression dataset and preprocessing*. Gene expression data were obtained from the Allen Human Brain Atlas (AHBA), which comprises over 20,000 genes assayed across 3702 tissue samples from six postmortem donors. Because right‐hemisphere sampling in the AHBA dataset is incomplete (only 4 of 6 donors provided right‐hemisphere data), we restricted all transcriptomic analyses to the left hemisphere, following established protocols [43, 44]. Accordingly, the regional expression matrix included 100 left‐hemisphere cortical parcels from the Schaefer‐200 atlas. Preprocessing was conducted using the abagen toolbox. The pipeline included: probe filtering (intensity > background in ≥ 50% of samples), selection of the most differentially stable probe per gene, registration of samples to the Schaefer‐200 atlas (2‐mm distance threshold), and donor‐wise z‐score normalization to control for inter‐individual differences.

*Association between gene expression and differential overlapping regions*. To determine which transcripts were linked to iTBS‐related alterations in overlapping architecture, we examined the correspondence between regional gene expression profiles and the brain areas showing differential overlap changes between the real and sham stimulation conditions. Specifically, gene expression profiles for the identified differential regions were obtained from the AHBA [44]. We then conducted cross‐regional Pearson correlations to examine the association between these expression values and the treatment‐related changes in H across groups. To address multiple comparisons and mitigate the influence of spatial autocorrelation, a two‐step significance testing framework was employed. First, for each gene, we computed a spatial autocorrelation‐corrected *p*‐value using a nonparametric spatial permutation test (10,000 rotations of the differential brain map via the spin_test function from the neuromaps toolbox). Rotations were hemisphere‐preserving on spherical surfaces; parcels overlapping the medial wall were handled by excluding them from the rotation procedure to maintain the empirical distance‐dependence. Second, we applied the FDR correction to these corrected *p*‐values (*p* < 0.05). This combined approach ensures that reported associations are robust against both multiple testing and spatial non‐independence.

*Disease‐related differential expression analysis*. To test the disorder specificity of the imaging‐derived gene associations, we extracted log_2_ fold‐change (log_2_FC) values from the cross‐disorder transcriptomic dataset of Gandal et al. [45]. covering ASD, major depressive disorder (MDD), schizophrenia (SCZ), bipolar disorder (BD), alcoholism and inflammatory bowel disease (IBD). For each disorder, Spearman's correlations were computed between the imaging–gene association coefficients of the identified genes and their corresponding log_2_FC values. Statistical significance was determined by 10,000 permutation tests with FDR correction (*p* < 0.05). A significant or oppositely directed correlation unique to a specific disorder was interpreted as evidence of disease‐associated transcriptional coupling.

*Gene enrichment analysis*. Genes implicated in the preceding association analyses were subjected to functional enrichment procedures. Functional annotation was first conducted using the ToppGene platform [46], an integrated toolset commonly employed for gene‐function characterization and candidate‐gene prioritization through annotation‐ and network‐based approaches [47]. To characterize the biological roles of these genes, Gene Ontology (GO) enrichment was performed across the domains of biological process, cellular component, and molecular function. Tissue‐, cell type‐, and developmental stage–specific expression patterns were then evaluated using the online Tissue Specific Expression Analysis (TSEA) resource [48] and the Cell Type‐Specific Expression Analysis (CTEA) tool [49, 50]. These analyses allowed us to determine the iTBS‐associated gene expression profiles within distinct tissues, cortical cell classes, and developmental periods. Specificity index probabilities (P^SI^ = 0.05, 0.01, and 0.001) were applied to quantify the likelihood that a gene showed selective expression within a given class [51].

*Protein–protein interaction (PPI) analysis*. PPI analyses were conducted using the STRING v12.0 [52] to investigate potential interactions among the associated genes. A PPI network was generated, and hub genes were defined as those falling within the top 30% of node‐degree rankings, with a minimum confidence score of 0.45. Furthermore, developmental expression patterns of the identified hub genes were assessed using data from the Human Brain Transcriptome Database [53, 54].

## 3 Results

### 3.1 Participant Characteristics

Table 1 presents the demographic characteristics of the participants (real iTBS and sham iTBS) at baseline. No significant differences existed between these 2 groups at baseline.

### 3.2 Clinical Outcome

Post‐treatment ISI scores were markedly lower in the real iTBS group relative to the sham condition (*p* = 0.007). A significant group × time interaction was observed for ISI improvement (*F*_(1,68)_ = 12.99, *p* = 0.001). Within‐group comparisons revealed a mean decrease of −6.94 in the real‐stimulation group (95% CI: −8.16 to −5.72; *p* < 0.001), whereas the sham group exhibited a smaller reduction of −3.54 (95% CI: −5.03 to −2.06; *p* < 0.001) (Figure S1).

### 3.3 Network Organization Differences Before and After iTBS

**FIGURE 2:**
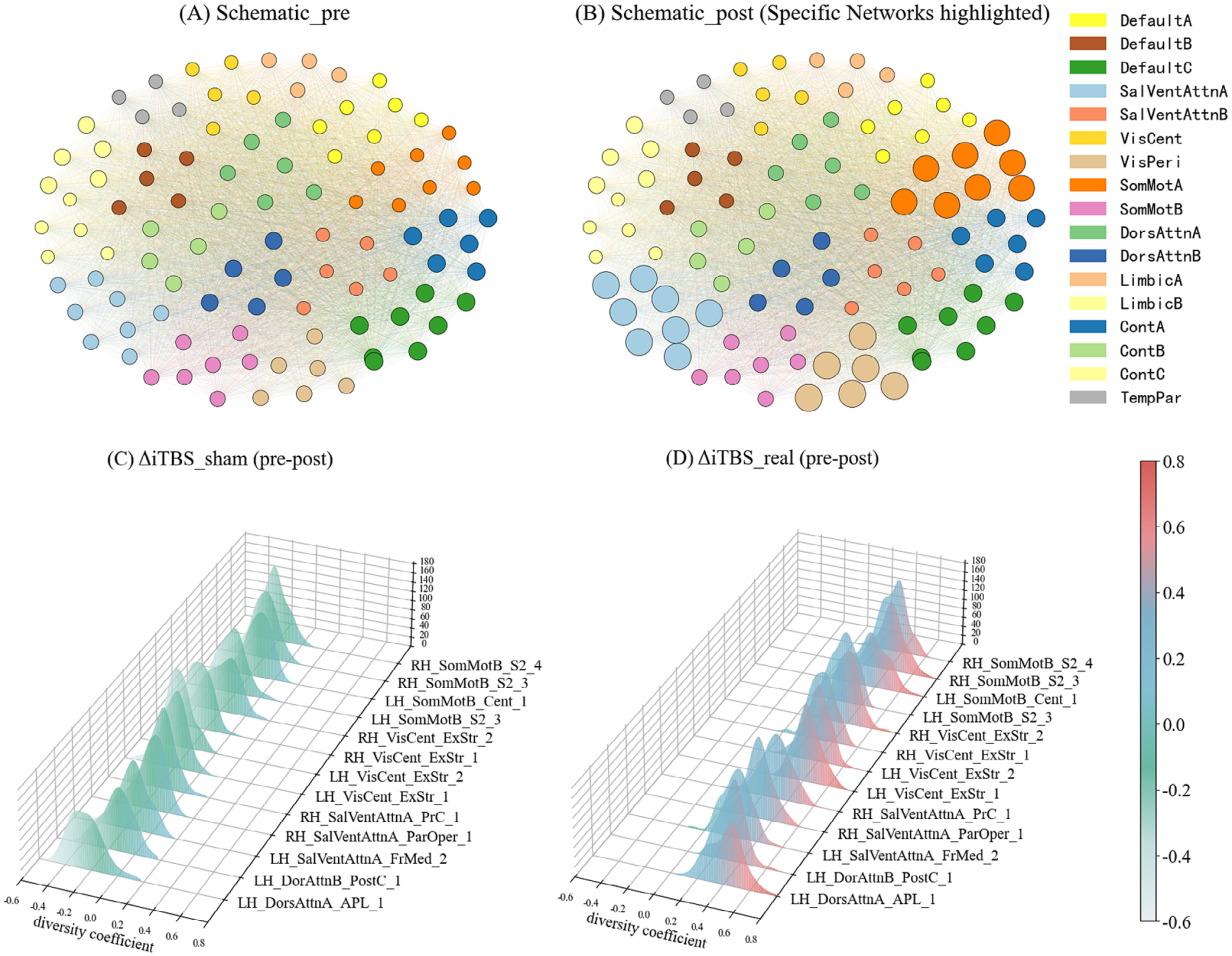
Schematic illustration of brain networks and pre‐ to post‐treatment changes in H. (A) Pretreatment schematic network configuration. (B) Post‐treatment schematic network configuration, with subnetworks containing significant iTBS‐related changes highlighted. The atlas comprises 200 cortical regions of interest assigned to 17 functional networks. Node colors correspond to the 17‐network parcellation shown in the legend. Larger nodes in panel B indicate cortical parcels showing significant group × time interaction effects in H. (C, D) Ridge plots showing the distribution of pre‐ to post‐treatment changes in H (ΔH) across the 13 significant cortical parcels in (C) the sham iTBS group and (D) the real iTBS group. H, Shannon‐entropy diversity coefficient.

As a preliminary step prior to analyzing the H, we reconstructed 17 large‐scale cortical networks, including the control (Cont A, B, C), default mode (Default A, B, C), dorsal attention (DorsAttn A, B), limbic (Limbic A, B), salience/ventral attention (SalVentAttn A, B), somatomotor (SomMot A, B), temporal–parietal (TempPar), visual central (VisCent), and visual peripheral (VisPeri) networks. To facilitate visualization, each network within the Schaefer‐200 cortical parcellation was color‐coded according to its spatial correspondence with the canonical 17‐network atlas. The network configurations in the real iTBS group before and after treatment were illustrated in Figure 2A,B. Relative to the sham group, participants receiving real iTBS exhibited marked alterations in functional connectivity (i.e., edges) spanning multiple cortical regions (i.e., nodes). In contrast, the sham iTBS group showed no appreciable differences in network configuration between the pre‐ and post‐treatment sessions.

### 3.4 Differences in Overlapping Brain Architecture Between Groups

To map alterations in overlapping system organization across the 17 large‐scale cortical networks, H were computed for every cortical region at both pre‐ and post‐treatment assessments in the real and sham iTBS groups. Group comparisons revealed 13 cortical regions that showed significant treatment‐related changes in the real iTBS group relative to the sham group (*p* < 0.05, FDR corrected). These regions were predominantly distributed within the attention network (*n* = 5), the somatomotor network (SMN, *n* = 4), and the visual network (*n* = 4) (Figure 2C,D). Detailed repeated‐measures ANOVA results, including *F* statistics and FDR‐corrected *p* values, were shown in Table S1.

### 3.5 Neuroimaging‐Clinical Association

Four regions showed significant associations between ΔH and ΔISI. These regions were mainly distributed in the visual central network, including the left VisCent_ExStr_1, left VisCent_ExStr_2, right VisCent_ExStr_1, and right VisCent_ExStr_2. Detailed partial correlation coefficients and *p* values are provided in Table S2.

### 3.6 Prediction of iTBS Using Baseline H

Baseline H showed preliminary predictive value for sleep improvement in the exploratory SVR analysis. The prediction exceeded chance level in permutation testing (observed *r* = 0.52, permutation *p* = 0.001; Figure S2).

### 3.7 Correlation Between ΔH and Neurotransmitter Profiles

**FIGURE 3:**
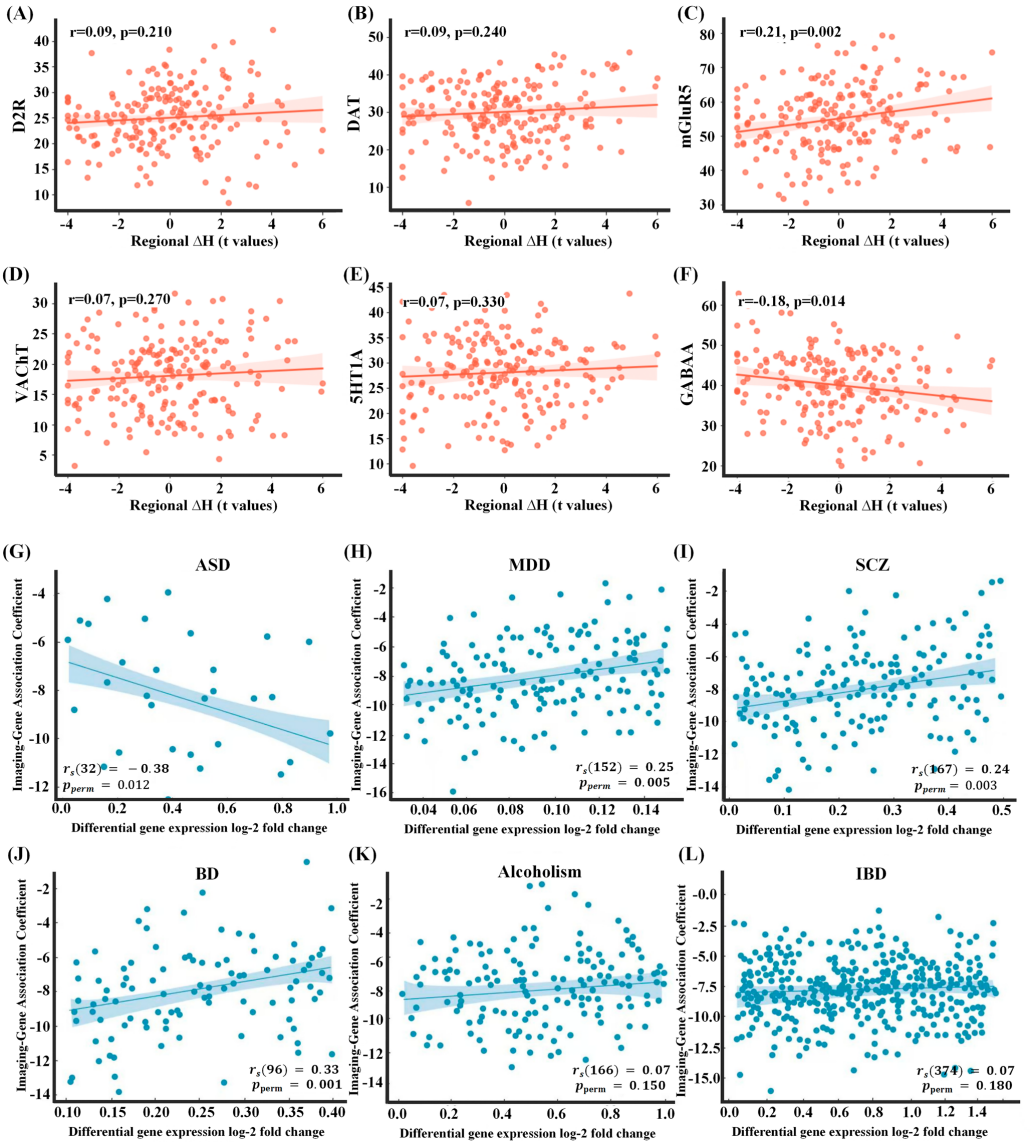
Neurotransmitter and transcriptomic associations of iTBS‐related H changes. (A‐F) Spatial correlations between regional ΔH t values and PET‐derived neurotransmitter maps for D2R, DAT, mGluR5, VAChT, 5‐HT1A, and GABA_A. Each point represents a cortical parcel. (G‐L) Correlations between imaging–gene association coefficients and disease‐related differential gene expression profiles for ASD, MDD, SCZ, BD, alcoholism, and IBD. Correlation coefficients and *p* values are shown in each panel. ASD, autism spectrum disorder; MDD, major depressive disorder; SCZ, schizophrenia; BD, bipolar disorder; IBD, inflammatory bowel disease; D2R, dopamine D2 receptor; DAT, dopamine transporter; mGluR5, metabotropic glutamate receptor 5; VAChT, vesicular acetylcholine transporter; 5‐HT1A, serotonin 1A receptor; GABA_A, gamma‐aminobutyric acid type A receptor.

The ΔH showed significant correlations with two neurotransmitter receptor profiles: mGluR5 (*r* = 0.22, *p* = 0.002, FDR‐corrected) and GABA_A (*r* = −0.18, *p* = 0.014, FDR‐corrected). No significant associations were observed for the remaining neurotransmitter systems, including D2R (*r* = 0.09, *p* = 0.210), DAT (*r* = 0.09, *p* = 0.240), VAChT (*r* = 0.074, *p* = 0.270) and 5HT1a (*r* = 0.07, *p* = 0.330) (Figure 3A–F).

### 3.8 Association Between ΔH and Gene Expression

For gene identification, normalized transcriptional data from more than 15,000 genes were obtained from 3121 tissue samples in the AHBA dataset following relabeling and refined probe selection. These data were then mapped onto cortical parcels to generate a left‐hemisphere regional expression matrix. This matrix was used to compute spatial correlations between gene expression and ΔH. In the real iTBS group, spatial correlation analyses identified 64 genes (Table S3) whose expression patterns significantly matched the spatial distribution of iTBS‐related changes in the overlapping brain system (*p* < 0.05, FDR corrected).

### 3.9 Disease‐Related Differential Expression Analysis Results

We identified 34 genes that were shared between our gene set and those reported by Gandal et al. [45] as significantly dysregulated in postmortem ASD case–control brain tissue. Moreover, the spatial association coefficients of these genes showed a significant negative correlation with their ASD‐related expression alterations (r_s(32)_ = −0.39, adjusted *p*_perm_ = 0.012, FDR‐corrected). This association showed a strong relevance to ASD, as it was not observed in the other five disorders: MDD (r_s(152)_ = 0.25, adjusted *p*_perm_ = 0.005, FDR‐corrected), SCZ (r_s(167)_ = 0.24, adjusted *p*_perm_ = 0.003, FDR‐corrected), BD (r_s(96)_ = 0.33, adjusted *p*_perm_ = 0.001, FDR‐corrected), alcoholism (r_s(166)_ = 0.06, adjusted *p*_perm_ = 0.150, FDR‐corrected), and IBD (r_s(374)_ = 0.07, adjusted *p*_perm_ = 0.180, FDR‐corrected) (Figure 3G–L).

### 3.10 Gene Functional Enrichment

**FIGURE 4:**
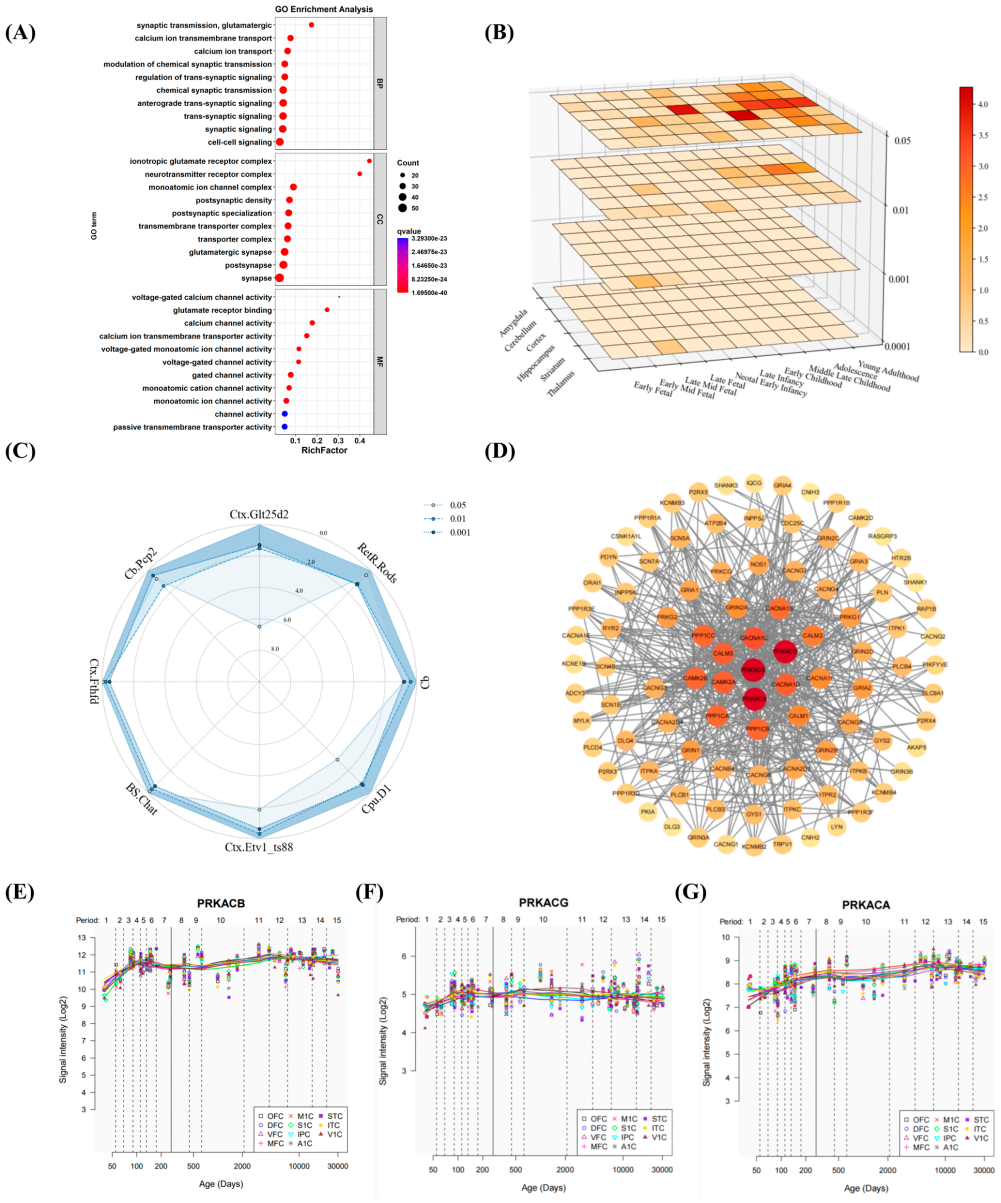
Gene enrichment and molecular characterization of genes associated with iTBS‐related H changes. (A) Gene Ontology enrichment analysis of the identified genes. (B) Spatiotemporal expression patterns of the identified gene set across brain regions and developmental periods. (C) Cell‐type specificity analysis across major cortical cell types. (D) Protein–protein interaction network of the identified gene set. Node color indicates network centrality, with PRKACB, PRKACG, and PRKACA identified as hub genes. (E–G) Developmental expression trajectories of representative hub genes, including PRKACB, PRKACG, and PRKACA. BP, biological process; CC, cellular component; MF, molecular function; H, Shannon‐entropy diversity coefficient; GO, Gene Ontology; PPI, protein–protein interaction.

To explore the biological significance of the iTBS‐sensitive gene set, we performed a comprehensive Gene Ontology (GO) enrichment analysis (Tables S4 and S5). The analysis revealed that the 64 identified genes were significantly enriched in key biological processes such as glutamatergic synaptic transmission and calcium ion transport; cellular components including the postsynaptic density and glutamatergic synapse; and molecular functions such as voltage‐gated calcium channel activity and glutamate receptor binding (FDR corrected, *p* < 0.05). These enrichment profiles highlight the strong involvement of synaptic and calcium‐related pathways within the iTBS‐sensitive gene set (Figure 4A).

### 3.11 Tissue, Cell Type, and Temporal‐Specific Expression

Temporal expression analysis revealed a distinct developmental pattern, characterized by elevated expression during mid‐to‐late childhood and adolescence, with secondary peaks in early infancy and young adulthood (Figure 4B). Tissue specificity analyses identified the brain as the primary site of expression for these genes. With the highest enrichment observed in cortical and cerebellar regions, followed by hippocampus and striatum. Cell type–specific profiling further demonstrated significant enrichment within neuronal populations, particularly cortical excitatory neurons (Ctx.Glt25d2) and cerebellar Purkinje cells (Cb.Pcp2), as well as in cholinergic systems across multiple brain regions (Figure 4C).

### 3.12 PPI Networks and Hub Genes

PPI analysis revealed a highly interconnected protein–protein interaction network (Figure 4D; Table S6). The resulting network contained 765 edges, significantly greater than the expected 60 edges (PPI enrichment *p*‐value < 1.0 × 10^−16^), indicating strong functional interconnectivity among these genes. The network exhibited a densely connected core module centered on PRKACB, PRKACA, and PRKACG, which form the catalytic subunits of the cAMP‐dependent protein kinase A (PKA) complex. These hub genes were closely linked to calcium channel genes (CACNA1C, CALM1, CAMK2A) and postsynaptic scaffold components (GRIN2B, DLG4). Spatiotemporal expression profiling further demonstrated that all three PRKA genes displayed stable cortical expression across lifespan (Figure 4E–G), with pronounced peaks during late childhood and adolescence.

## 4 Discussion

This study demonstrated that an eight‐week course of neuronavigated iTBS over the left OFC induced spatial reorganization of overlapping system‐level architecture in ASD with insomnia. The most prominent alterations occurred in the attention, somatomotor, and visual networks and were associated with improvements in insomnia severity. JuSpace analysis revealed that these changes spatially corresponded to regional mGluR5 and GABA_A receptor densities, implicating E/I‐related chemoarchitecture. Transcriptomic profiles further identified convergent gene‐expression patterns across iTBS‐sensitive regions. To our knowledge, this study is among the first to integrate neuroimaging, neurotransmitter mapping, and transcriptomic analyses within the framework of overlapping system‐level architecture, providing a multiscale perspective on the biological context underlying iTBS‐related network modulation in ASD with insomnia.

iTBS‐related changes in cross‐module participation were mainly observed in the attention network, SMN, and visual network. One possible explanation is that the observed effects reflect the broad functional connectivity of the OFC. The OFC is functionally connected with multiple cortical networks, including the attention network [55], SMN [56], and visual network [57]. Therefore, iTBS over the OFC may influence distant cortical networks through these functional connections. This interpretation may help explain why changes in H were detected across multiple cortical networks rather than being restricted to the stimulated region. A second, more indirect route may involve cortico‐subcortical circuitry. The OFC is part of prefrontal cortico‐striato‐thalamo‐cortical loops, linking cortical regions with the striatum and thalamus before returning to the prefrontal cortex. Through these loops, stimulation‐related effects could potentially influence broader cortical dynamics via subcortical relay pathways. Such a possibility is relevant to ASD with insomnia because cortico‐subcortical circuits are involved in sensory processing [58], and sleep‐related functions [59]. However, this interpretation remains indirect. The present study examined cortical functional networks and did not directly measure subcortical activity, structural pathways, or cortico‐subcortical coupling. Future studies using high‐resolution fMRI, diffusion imaging, or multimodal stimulation‐imaging designs are needed to test whether these pathways contribute to treatment‐related changes in cross‐module participation.

Our findings showed spatial associations between H and two major neurochemical systems, with a positive association for mGluR5 and a negative association for GABA_A receptors. Specifically, regions with greater H tended to align with regions showing higher mGluR5 receptor availability but lower GABA_A receptor availability. This pattern may provide a neurochemical context for understanding functional network integration in ASD, particularly from the perspective of excitation–inhibition balance. mGluR5‐related pathways have been implicated in synaptic dysfunction and neurodevelopmental mechanisms relevant to ASD. In fragile X syndrome, a major monogenic form of ASD, enhanced mGluR5 pathway activity has been proposed as an important disease‐related mechanism [60]. In parallel, preclinical evidence showing that mGluR5‐directed treatment can reverse functional connectivity deficits further supports the potential translational relevance of the mGluR5–H association [61]. Experimental evidence has also shown that GABA_A receptor abundance is involved in synaptic connectivity and neuronal wiring, with relevance to excitation–inhibition imbalance in neuropsychiatric disorders [62]. Together, these findings suggest that the balance between glutamatergic and GABAergic systems may be relevant to individual differences in cross‐module functional participation. Our results may provide a basis for future studies examining whether glutamatergic and GABAergic pathways contribute to altered network connectivity in ASD and whether these pathways are relevant to stimulation‐related changes in functional network integration in ASD.

It is important to emphasize that although the spatial correlations between H and neurotransmitter receptor densities were statistically significant, the observed effect sizes were modest. This indicates that chemoarchitecture may provide one relevant spatial scaffold for iTBS‐related network reorganization, but it accounts for only part of the observed spatial variance. Additional neurobiological factors, such as white matter integrity, local microcircuit architecture, and individual baseline cortical excitability, may interact with receptor distributions to shape the final topographical pattern of network reorganization. Thus, the neurotransmitter findings should be interpreted as spatial correspondence evidence rather than direct proof of receptor‐level modulation in individual participants.

Our transcriptomic analysis further links these macroscale neuroimaging findings to a molecular context. The iTBS‐sensitive regions were enriched for a co‐expressed gene set functionally associated with synaptic signaling and voltage‐gated calcium channel activity. The core protein–protein interaction network of this gene set converged on a Ca^2+^–PKA signaling axis, including CACNA1C, CALM1, and PRKACB/G. This pattern suggests a potential molecular context through which stimulation‐related network changes may be biologically situated. These findings do not demonstrate direct gene‐expression changes after iTBS. Rather, they suggest that iTBS‐sensitive cortical regions are spatially embedded within a normative transcriptomic context related to calcium‐dependent synaptic signaling and plasticity [63, 64, 65]. The developmental timing of this gene set further supports its relevance. Our analysis showed that these genes exhibit prominent expression during late childhood and adolescence, a critical period for experience‐dependent synaptic pruning, circuit refinement, and large‐scale network maturation. This developmental trajectory aligns with large‐scale transcriptomic evidence [66] and structural co‐variation analyses [67], suggesting that the identified gene program may reflect an endogenous molecular context associated with heightened cortical plasticity. Previous studies have also suggested that brain stimulation may influence epigenetic and gene‐expression‐related processes [68]. In this context, iTBS may interact with developmentally sensitive plasticity‐related pathways, although this possibility remains hypothetical and requires direct experimental validation. Future studies combining neuroimaging with participant‐level molecular, epigenetic, or peripheral biomarker data are needed to determine whether similar mechanisms are preserved or disrupted in neurodevelopmental conditions such as ASD with comorbid insomnia.

From a clinical perspective, the observed iTBS‐related changes in H within the attention network, SMN, and visual network may provide preliminary translational insights. First, iTBS‐related H changes were statistically significant across the 13 identified cortical regions (all FDR‐corrected *p* < 0.05). Among these regions, four visual‐network parcels showed significant associations between ΔH and insomnia symptom reduction after FDR correction. This quantitative association suggests that Shannon‐entropy diversity may provide a candidate imaging feature for future studies of treatment monitoring. Second, using baseline H values from these four clinically relevant visual regions, our exploratory SVR analysis predicted subsequent ISI improvement above chance level. This finding raises the possibility that baseline cross‐module participation may help inform future patient‐stratification models and identify individuals who are more likely to benefit from iTBS. However, this interpretation remains preliminary and requires validation in larger independent samples. The translational relevance of these findings may extend beyond sleep regulation alone. Sleep disturbance is common in ASD and may exacerbate autism‐related difficulties, including sensory dysregulation, irritability, repetitive behaviors, and social communication problems, with recent evidence linking sleep disruption to greater overall ASD symptom burden [1, 2, 3]. In this context, the significant association between iTBS‐related visual‐network H changes and insomnia improvement raises the possibility that sleep‐related imaging changes may be relevant to broader ASD symptomatology. Future longitudinal studies should directly examine whether these changes are associated with measurable improvements in daytime functioning, social communication, and repetitive behaviors. If replicated, these findings may help clarify whether sleep‐related changes in cross‐module participation represent one pathway through which iTBS is linked to broader clinical outcomes in ASD with comorbid insomnia.

### 4.1 Limitations and Future Directions

Several limitations should be acknowledged. First, because no neurotypical comparison group or comparable external benchmark was available, we could not determine whether the post‐iTBS changes moved toward neurotypical levels of overlapping network organization. Therefore, the present findings should be interpreted cautiously as treatment‐related reorganization of overlapping cortical network architecture, rather than normalization of brain network function. Second, the SVR findings should be considered preliminary and exploratory. Although baseline H values showed potential for predicting insomnia improvement, LOOCV in a modest sample may yield unstable or optimistic performance estimates. Larger independent cohorts are needed to test the robustness and generalizability of this predictive model. Third, although the association between iTBS‐related network reorganization and insomnia improvement supports the clinical relevance of our findings, detailed measures of daytime functioning and arousal were unavailable. Therefore, we could not determine whether sleep improvement was accompanied by broader changes in ASD‐related symptoms or behavioral functioning. Future studies should include multidimensional clinical assessments to clarify the functional significance of sleep‐related network reorganization. In addition, current pharmacological options for ASD‐related insomnia remain supported by limited and low‐certainty evidence, while hypnotic medications are already widely used in pediatric ASD and other neurodevelopmental populations. These clinical challenges highlight the need for safer and biologically informed interventions. In this context, iTBS may represent a promising non‐pharmacological strategy, although larger trials are needed to confirm its efficacy, safety, and long‐term clinical utility. Finally, future studies should combine longitudinal neuroimaging, individualized electric‐field modeling, and participant‐level molecular or physiological measurements to clarify how stimulation parameters, baseline neurobiology, and network topology jointly determine treatment response.

## 5 Conclusions

These findings highlight overlapping network reorganization as a clinically relevant process associated with sleep improvement in ASD with chronic insomnia, and provide neurobiological insight into mGluR5/GABA_A‐ and Ca^2+^‐PKA‐related pathways that may inform biomarker‐guided iTBS strategies.

## Author Contributions

Conceptualization: Huashuang Zhang. Study design: Huashuang Zhang and Bincan Xiong. Methodology: Huashuang Zhang, Bincan Xiong, Hongchi Liu, and Jingwen Zhang. Data collection: Yilin Huang, Ruimin Yu and Wenqi Jiang. Statistical analysis: Bincan Xiong and Hongchi Liu. Writing‐original draft: Huashuang Zhang. Writing‐review and editing: Huashuang Zhang, Bincan Xiong, Yilin Huang and Tongjia She. Funding acquisition: Huashuang Zhang, Bincan Xiong, and Ruimin Yu. All authors approved the final manuscript.

## Funding

This work was supported by the Research Cultivation Program of The Third Affiliated Hospital, School of Medicine, Foshan University (grant number FUTH2026C008), Specialized Research Funding for the Technology Innovation of Foshan (grant number 2420001004544); Foshan University High‐level Talent Start‐up Funding (grant number CGZ07508) and Foshan University Student Academic Funding (grant numbers xsjj202519zrc11, xsjj202519zrb03).

## Ethics Statement

The trial protocol was approved by the Ethics Committee of Foshan University (approval ID: FUME2024016). This study was registered with the Chinese Clinical Trial Registry (ChiCTR2600116100).

## Conflicts of Interest

The authors declare no conflicts of interest.

## Supporting information


**Data S1:** cns71041‐sup‐0001‐Supinfo01.docx.

**Figure S1:** Clinical outcome result.
**Figure S2:** SVR prediction result.
**Table S1:** Imaging results for the 13 differential overlapping regions.
**Table S2:** Network‐clinical association.
**Table S3:** 64 genes were significantly spatially correlated with the differential brain overlapping system in the real iTBS group.
**Table S4:** Functional enrichment results of the genes associated with real iTBS.
**Table S5:** Gene enrichment analysis: gene‐pathway mapping.
|**Table S6:** The details of the 64 hub genes in the interconnected PPI network.

## Data Availability

PET‐derived neurotransmitter distribution maps are accessible via the JuSpace Toolbox (https://github.com/juryxy/JuSpace) within MATLAB R2024b. Postmortem gene expression profiles are available from the Allen Human Brain Atlas (https://help.brain‐map.org). Gene expression data from the Allen Human Brain Atlas were preprocessed using the abagen toolbox (https://github.com/rmarkello/abagen) within Python 3.12.7. Gene enrichment analysis was performed using Metascape (https://metascape.org/gp/index.html). Brain mapping images were visualized with MRIcron (https://www.nitrc.org/projects/mricron). All data within the study are presented in the paper and/or the Supporting Information. The original data generated and/or analyzed during the current study are available from the corresponding author upon reasonable request. Due to the sensitive nature of the clinical data and restrictions of informed consent, participant‐level data are not publicly available.
