# Supplementary material for: Intermittent Theta Burst Stimulation Improves Sleep in Autism Spectrum Disorder by Reorganizing Overlapping Brain Networks With Neurochemical and Transcriptomic Signatures: A Randomized Controlled Trial
Source: CNS Neurosci Ther. 2026 Aug 12;32(8):e71041. doi: 10.1002/cns.71041 (PMC13469180; doi:10.1002/cns.71041)
Supplement: Supplementary file 2 [file CNS-32-e71041-s002.docx]

**Supporting Information Content**

Intermittent Theta Burst Stimulation Improves Sleep in Autism Spectrum Disorder by Reorganizing Overlapping Brain Networks with Neurochemical and Transcriptomic Signatures: A Randomized Controlled Trial

**Method:** Supplementary methods for navigation.

**Figure S1.** Clinical outcome result.

**Figure S2.** SVR prediction result.

**Table S1.** Imaging results for the 13 differential overlapping regions.

**Table S2.** Network-clinical association.

**Table S3.** 64 genes were significantly spatially correlated with the differential brain overlapping system in the real iTBS group.

**Table S4.** Functional enrichment results of the genes associated with real iTBS.

**Table S5**. Gene enrichment analysis: gene-pathway mapping.

**Table S6.** The details of the 64 hub genes in the interconnected PPI network.

This supplementary material has been provided by the authors to give readers additional information about their work.

**Supplementary methods for navigation**

To enhance anatomical precision and minimize interindividual variability, neuronavigation was implemented using the Navigator software, enabling stereotaxic registration of each participant’s brain with the TMS coil (Julkunen et al., 2009; Ruohonen et al., 2010). During baseline or sham sessions (conducted prior to MPRAGE acquisition), a standardized MNI template guided the navigation, whereas for the active iTBS session, each participant’s individual MPRAGE image was employed to ensure maximal anatomical accuracy.

The left OFC stimulation site was localized in three dimensions following well-established anatomical landmark protocols (Crespo-Facorro et al., 1999). Initially, the EEG International 10–20 system was used to identify position Fp1, a procedure that compensates for variations in skull size and is routinely applied in clinical TMS when neuronavigation is unavailable. The coil was first positioned tangentially to the scalp at Fp1, and the resulting focal point of stimulation was visualized on the participant’s structural MRI within the neuronavigation system. A digital marker was then placed at this focal point, and its coordinates were verified to ensure that they corresponded to the intended left OFC/frontopolar cortex (Brodmann area 10). If the Fp1-based position did not accurately align with the target region, the coil was gradually adjusted inferiorly and/or laterally until the projected stimulation site was located within Brodmann area 10.

The neuronavigation procedure aimed to deliver stimulation to BA10 as close as possible to the adjacent BA11. However, precise placement in more ventral regions was physically constrained by the geometry of the coil and individual head morphology (e.g., forehead curvature and nasal bridge distance). Consequently, the final scalp positions and MNI coordinates of the stimulation foci showed slight interindividual variation. Nevertheless, across participants, the focal point consistently localized just superior to the left eye, near or over the brow line. Sensitivity analyses confirmed that these small coordinate variations had negligible influence on neural or behavioral results (see Neuronavigational target coordinates section below).

**Neuronavigational target coordinates**

For each participant, the neuronavigation system provided the three-dimensional MNI coordinates of the stimulation focal point during the active iTBS session. To evaluate whether individual differences in stimulation loci contributed to outcome variability, we correlated the x-, y-, and z-coordinates with post-treatment changes in the Shannon-entropy diversity coefficient (ΔH) and ISI scores (ΔISI), analyzed separately for the iTBS and sham groups.

No systematic or spatially consistent associations were observed between target coordinates and either neural or behavioral outcomes. Coordinate variability in any axis (x, y, z) did not significantly relate to post-treatment H values or their change from baseline, nor to ΔISI scores in either group. These findings indicate that **minor deviations in the neuronavigational focal point had negligible influence on both network-level entropy measures and behavioral performance**, confirming the spatial robustness of the left OFC targeting. Moreover, the high reproducibility of results across participants supports the reliability of the iTBS stimulation protocol and suggests that observed treatment effects primarily reflect neurophysiological modulation rather than geometric variability in coil placement.

**References**

Julkunen P, Säisänen L, Danner N, Niskanen E, Hukkanen T, Mervaala E, Könönen M: Comparison of navigated and non-navigated transcranial magnetic stimulation for motor cortex mapping, motor threshold and motor evoked potentials. Neuroimage 2009;44(3):790-5

Ruohonen J, Karhu J: Navigated transcranial magnetic stimulation. Clin Neurophysiol 2010;40(1):7-17

Crespo-Facorro B, Kim JJ, Andreasen NC, O'Leary DS, Wiser AK, Bailey JM, Harris G, Magnotta VA: Human frontal cortex: an MRI-based parcellation method. Neuroimage 1999;10(5):500-19

**Figure S1.** Clinical outcome result. Group-level changes in Insomnia Severity Index (ISI) scores before and after treatment, showing a significantly greater reduction in the real iTBS group compared with the sham group. iTBS, intermittent theta-burst stimulation ; ISI, Insomnia Severity Index.

(A)

**Figure S2.** SVR prediction result. (A) Scatter plot showing the relationship between SVR-predicted and actual ISI improvement, demonstrating significant predictive performance (r = 0.52). (B) Permutation-based null distribution (1,000 iterations) of correlation coefficients, with the red line marking the true model estimate, indicating that the prediction accuracy was unlikely due to chance (*p* = 0.001). SVR, Support vector regression.

| **Table S1.** Imaging results for the 13 differential overlapping regions. | | | | | | | | |
| --- | --- | --- | --- | --- | --- | --- | --- | --- |
| **Region label** | **Full region name** | **Hemisphere** | **Network** | **F** | Uncorrected p | FDR-corrected p | **ΔH real, mean ± SD** | **ΔH sham, mean ± SD** |
| LH_DorsAttnA_APL_1 | Left anterior parietal lobule, dorsal attention network A | Left | Dorsal Attention | 38.72 | <0.001 | <0.001 | 0.168 ± 0.136 | 0.021 ± 0.118 |
| LH_DorsAttnB_PostC_1 | Left postcentral region, dorsal attention network B | Left | Dorsal Attention | 29.45 | <0.001 | <0.001 | 0.139 ± 0.121 | -0.006 ± 0.107 |
| LH_SalVentAttnA_FrMed_2 | Left medial frontal region, salience/ventral attention network A | Left | Salience/Ventral attention | 16.83 | <0.001 | 0.008 | 0.094 ± 0.113 | 0.018 ± 0.099 |
| RH_SalVentAttnA_ParOper_1 | Right parietal opercular region, salience/ventral attention network A | Right | Salience/Ventral attention | 25.96 | <0.001 | 0.002 | 0.132 ± 0.129 | -0.014 ± 0.115 |
| RH_SalVentAttnA_PrC_1 | Right precentral region, salience/ventral attention network A | Right | Salience/Ventral attention | 11.37 | 0.001 | 0.031 | 0.076 ± 0.105 | 0.025 ± 0.096 |
| LH_VisCent_ExStr_1 | Left extrastriate region, central visual network | Left | Visual central | 23.18 | <0.001 | 0.003 | 0.121 ± 0.124 | -0.011 ± 0.109 |
| LH_VisCent_ExStr_2 | Left extrastriate region, central visual network | Left | Visual central | 14.69 | <0.001 | 0.016 | 0.086 ± 0.112 | 0.014 ± 0.101 |
| RH_VisCent_ExStr_1 | Right extrastriate region, central visual network | Right | Visual central | 20.54 | <0.001 | 0.005 | 0.109 ± 0.119 | -0.019 ± 0.112 |
| RH_VisCent_ExStr_2 | Right extrastriate region, central visual network | Right | Visual central | 13.21 | 0.001 | 0.024 | 0.081 ± 0.108 | 0.031 ± 0.097 |
| LH_SomMotB_S2_3 | Left secondary somatosensory region, somatomotor network B | Left | Somatomotor | 34.88 | <0.001 | <0.001 | 0.154 ± 0.133 | -0.023 ± 0.119 |
| LH_SomMotB_Cent_1 | Left central somatomotor region, somatomotor network B | Left | Somatomotor | 27.62 | <0.001 | 0.001 | 0.126 ± 0.127 | 0.007 ± 0.106 |
| RH_SomMotB_S2_3 | Right secondary somatosensory region, somatomotor network B | Right | Somatomotor | 18.91 | <0.001 | 0.007 | 0.103 ± 0.116 | -0.016 ± 0.104 |
| RH_SomMotB_S2_4 | Right secondary somatosensory region, somatomotor network B | Right | Somatomotor | 10.82 | 0.002 | 0.036 | 0.069 ± 0.101 | 0.022 ± 0.093 |

| **Table S2.** Neuroimaging-clinical association. | | | |
| --- | --- | --- | --- |
| Network | Partial r | *p* value | *p* value, FDR-corrected |
| Visual central | -0.56 | 0.001 | 0.010 |
| Visual central | -0.51 | 0.003 | 0.020 |
| Visual central | -0.48 | 0.007 | 0.030 |
| Visual central | -0.44 | 0.015 | 0.049 |

**Table S3.** 64 genes were significantly spatially correlated with the differential brain overlapping system in the real iTBS group.

| **Identified Genes** | | | |
| --- | --- | --- | --- |
| AKAP5 | CACNA1C | CACNA1D | CACNA1F |
| CACNA1S | CACNA2D1 | CACNA2D4 | CACNB3 |
| CACNB4 | CACNG2 | CACNG3 | CACNG4 |
| CACNG6 | CACNG7 | CACNG8 | CALM1 |
| CALM2 | CALM3 | CAMK2A | CAMK2B |
| CNIH2 | CNIH3 | DGKZ | DLG1 |
| DLG3 | DLG4 | DLGAP1 | GRIA1 |
| GRIA2 | GRIA3 | GRIA4 | GRID1 |
| GRIN1 | GRIN2A | GRIN2B | GRIN2C |
| GRIN2D | GRIN3A | GRIN3B | HSPA2 |
| INPP5A | IPMK | ITPKA | KIF13B |
| KIF5A | KIF5B | KIF5C | LYN |
| MINPP1 | NOS1 | PIKFYVE | PIP4K2A |
| PLCB4 | PPFIA2 | PPP1CA | PPP1CB |
| PPP1CC | PRKACA | PRKACB | PRKACG |
| RASGRP3 | SHANK1 | SHANK2 | SHANK3 |

| **Table S4.** Functional enrichment results of the genes associated with real iTBS. | | | |
| --- | --- | --- | --- |
| **Category** | **Pathway** | ***p*-value** | ***p*-value FDR-corrected** |
| GO: Biological Process | synaptic transmission, glutamatergic | 1.26E-27 | 7.76E-25 |
| GO: Biological Process | calcium ion transmembrane transport | 7.53E-19 | 1.99E-16 |
| GO: Biological Process | calcium ion transport | 3.09E-17 | 7.41E-15 |
| GO: Biological Process | modulation of chemical synaptic transmission | 6.39E-20 | 1.87E-17 |
| GO: Biological Process | regulation of trans‑synaptic signaling | 6.39E-20 | 1.87E-17 |
| GO: Biological Process | chemical synaptic transmission | 1.26E-27 | 7.76E-25 |
| GO: Biological Process | anterograde trans‑synaptic signaling | 1.26E-27 | 7.76E-25 |
| GO: Biological Process | trans‑synaptic signaling | 1.26E-27 | 7.76E-25 |
| GO: Biological Process | synaptic signaling | 1.26E-27 | 7.76E-25 |
| GO: Biological Process | cell‑cell signaling | 1.61E-14 | 3.02E-12 |
| GO: Cellular Component | ionotropic glutamate receptor complex | 2.88E-29 | 2.15E-26 |
| GO: Cellular Component | neurotransmitter receptor complex | 2.88E-29 | 2.15E-26 |
| GO: Cellular Component | monoatomic ion channel complex | 5.60E-16 | 1.26E-13 |
| GO: Cellular Component | postsynaptic density | 5.24E-22 | 1.90E-19 |
| GO: Cellular Component | postsynaptic specialization | 5.24E-22 | 1.90E-19 |
| GO: Cellular Component | transmembrane transporter complex | 9.91E-19 | 2.58E-16 |
| GO: Cellular Component | transporter complex | 7.08E-14 | 1.21E-11 |
| GO: Cellular Component | glutamatergic synapse | 1.33E-19 | 3.63E-17 |
| GO: Cellular Component | postsynapse | 2.54E-11 | 3.55E-09 |
| GO: Cellular Component | synapse | 2.54E-11 | 3.55E-09 |
| GO: Molecular Function | voltage‑gated calcium channel activity | 3.02E-30 | 1.09E-27 |
| GO: Molecular Function | glutamate receptor binding | 1.14E-13 | 4.79E-11 |
| GO: Molecular Function | calcium channel activity | 1.07E-15 | 1.19E-13 |
| GO: Molecular Function | calcium ion transmembrane transporter activity | 1.74E-13 | 6.61E-11 |
| GO: Molecular Function | voltage‑gated monoatomic ion channel activity | 5.13E-13 | 1.74E-10 |
| GO: Molecular Function | voltage‑gated channel activity | 1.62E-12 | 5.01E-10 |
| GO: Molecular Function | gated channel activity | 4.68E-09 | 3.16E-07 |
| GO: Molecular Function | monoatomic cation channel activity | 5.49E-10 | 4.17E-08 |
| GO: Molecular Function | monoatomic ion channel activity | 1.82E-09 | 1.32E-07 |
| GO: Molecular Function | channel activity | 1.12E-09 | 7.76E-08 |
| GO: Molecular Function | passive transmembrane transporter activity | 4.17E-09 | 2.82E-07 |

| **Table S5.** Gene enrichment analysis: gene-pathway mapping. | | |
| --- | --- | --- |
| **Category** | **Pathway** | **Gene symbol** |
| GO: Biological Process | synaptic transmission, glutamatergic | CACNB4,GRIA1,GRIA2,GRIA3,GRIA4,GRID1,GRIN2A,GRIN2B,GRIN2C,GRIN2D,GRIN3A,GRIN3B,CNIH3,CNIH2 |
| GO: Biological Process | calcium ion transmembrane transport | CACNA1C,CACNA1D,CACNA1F,CACNA1S,CACNA2D1,CACNB3,CACNB4,GRIN1,GRIN2A,GRIN2B,GRIN2C,GRIN2D,PLCB4,CACNG3,CACNG2,CACNG4,CACNG8,CACNG7,CACNG6,CACNA2D4,GRIN3A,GRIN3B |
| GO: Biological Process | calcium ion transport | CACNA1C,CACNA1D,CACNA1F,CACNA1S,CACNA2D1,CACNB3,CACNB4,CAMK2A,GRIN1,GRIN2A,GRIN2B,GRIN2C,GRIN2D,PLCB4,CACNG3,CACNG2,CACNG4,CACNG8,CACNG7,CACNG6,CACNA2D4,GRIN3A,GRIN3B |
| GO: Biological Process | modulation of chemical synaptic transmission | CACNB4,CALM1,CALM2,CALM3,CAMK2A,CAMK2B,DLG4,GRIA1,GRIA2,GRIA3,GRIA4,GRID1,GRIN1,GRIN2A,GRIN2B,GRIN2C,GRIN2D,ITPKA,KIF5B,PRKACA,PPFIA2,DLGAP1,AKAP5,CACNG3,CACNG2,SHANK2,CACNG4,SHANK1,CACNG8,CACNG7,SHANK3,GRIN3A,GRIN3B |
| GO: Biological Process | regulation of trans‑synaptic signaling | CACNB4,CALM1,CALM2,CALM3,CAMK2A,CAMK2B,DLG4,GRIA1,GRIA2,GRIA3,GRIA4,GRID1,GRIN1,GRIN2A,GRIN2B,GRIN2C,GRIN2D,ITPKA,KIF5B,PRKACA,PPFIA2,DLGAP1,AKAP5,CACNG3,CACNG2,SHANK2,CACNG4,SHANK1,CACNG8,CACNG7,SHANK3,GRIN3A,GRIN3B |
| GO: Biological Process | chemical synaptic transmission | CACNB3,CACNB4,DLG1,DLG3,DLG4,GRIA1,GRIA2,GRIA3,GRIA4,GRID1,GRIN1,GRIN2A,GRIN2B,GRIN2C,GRIN2D,KIF5A,DLGAP1,AKAP5,GRIN3A,GRIN3B,CNIH3,CNIH2 |
| GO: Biological Process | anterograde trans‑synaptic signaling | CACNB3,CACNB4,DLG1,DLG3,DLG4,GRIA1,GRIA2,GRIA3,GRIA4,GRID1,GRIN1,GRIN2A,GRIN2B,GRIN2C,GRIN2D,KIF5A,DLGAP1,AKAP5,GRIN3A,GRIN3B,CNIH3,CNIH2 |
| GO: Biological Process | trans‑synaptic signaling | CACNB3,CACNB4,DLG1,DLG3,DLG4,GRIA1,GRIA2,GRIA3,GRIA4,GRID1,GRIN1,GRIN2A,GRIN2B,GRIN2C,GRIN2D,KIF5A,DLGAP1,AKAP5,GRIN3A,GRIN3B,CNIH3,CNIH2 |
| GO: Biological Process | synaptic signaling | CACNB3,CACNB4,DLG1,DLG3,DLG4,GRIA1,GRIA2,GRIA3,GRIA4,GRID1,GRIN1,GRIN2A,GRIN2B,GRIN2C,GRIN2D,KIF5A,NOS1,DLGAP1,AKAP5,GRIN3A,GRIN3B,CNIH3,CNIH2 |
| GO: Biological Process | cell‑cell signaling | CACNA1C,CACNA1D,CACNA1S,CACNA2D1,CACNB3,CACNB4,CALM1,CALM2,CALM3,CAMK2B,DLG1,DLG4,GRIA1,GRIN1,GRIN2A,GRIN2B,GRIN2C,GRIN2D,NOS1,PRKACA,SHANK1,SHANK3,CACNA2D4 |
| GO: Cellular Component | ionotropic glutamate receptor complex | DLG4,GRIA1,GRIA2,GRIA3,GRIA4,GRID1,GRIN1,GRIN2A,GRIN2B,GRIN2C,GRIN2D,SHANK1,GRIN3A,GRIN3B |
| GO: Cellular Component | neurotransmitter receptor complex | DLG4,GRIA1,GRIA2,GRIA3,GRIA4,GRID1,GRIN1,GRIN2A,GRIN2B,GRIN2C,GRIN2D,SHANK1,GRIN3A,GRIN3B |
| GO: Cellular Component | Monoatomic ion channel complex | CACNA1C,CACNA1D,CACNA1F,CACNA1S,CACNA2D1,CACNB3,GRIN1,GRIN2A,GRIN2B,GRIN2C,GRIN2D,PLCB4 |
| GO: Cellular Component | postsynaptic density | DLG1,DLG3,DLG4,GRIN2A,GRIN2C,KIF5A,KIF5B,KIF5C,CACNG3,CACNG2,SHANK1,CACNG7,CNIH3 |
| GO: Cellular Component | postsynaptic specialization | DLG1,DLG3,DLG4,KIF5A,KIF5B,KIF5C,CACNG3,CACNG2,CACNG4,CACNG8,CACNG7,CNIH3 |
| GO: Cellular Component | transmembrane transporter complex | CACNA1F,CACNB3,CALM1,CALM2,CALM3,CACNG3,CACNG2,CACNG4,CACNG8,CACNG7,CNIH3,CNIH2 |
| GO: Cellular Component | transporter complex | CACNB3,CALM3,DLG1,DLG3,DLG4,GRIN2A,GRIN2C,CACNG3,CACNG2,CACNG4,CACNG8,CACNG7,SHANK3,GRIN3B,CNIH3,PIKFYVE |
| GO: Cellular Component | glutamatergic synapse | CACNA1C,CACNA1D,DLG4,GRIA1,GRIA2,GRIA3,GRIA4,GRIN1,GRIN2A,GRIN2B,GRIN2C,GRIN2D,PLCB4,PRKACA,PRKACB,PRKACG,DLGAP1,SHANK2,SHANK1,SHANK3,GRIN3A,GRIN3B |
| GO: Cellular Component | postsynapse | CACNA1S,CACNB4,DLG1,DLG4,GRIA1,ITPKA,PPFIA2,AKAP5,CACNG2,SHANK2,SHANK1,SHANK3 |
| GO: Cellular Component | synapse | CACNA1S,CACNB4,DLG1,DLG4,GRIA1,ITPKA,PPFIA2,AKAP5,CACNG2,SHANK2,SHANK1,SHANK3 |
| GO: Molecular Function | voltage‑gated calcium channel activity | CACNA1C,CACNA1D,CACNA1F,CACNA1S,CACNA2D1,CACNB3,CACNB4 |
| GO: Molecular Function | glutamate receptor binding | DLG4,GRIA1,GRIA2,GRIA3,GRIA4,GRID1,GRIN1,GRIN2A,GRIN2B,GRIN2C,GRIN2D,SHANK1,GRIN3A,GRIN3B |
| GO: Molecular Function | calcium channel activity | CACNA1C,CACNA1D,CACNA1F,CACNA1S,CACNA2D1,CACNB3,CACNB4 |
| GO: Molecular Function | calcium ion transmembrane transporter activity | CACNA1C,CACNA1D,CACNA1F,CACNA1S,CACNA2D1,CACNB3,CACNB4,GRIN1,GRIN2A,GRIN2B,GRIN2C,GRIN2D |
| GO: Molecular Function | voltage‑gated monoatomic ion channel activity | CACNA1C,CACNA1D,CACNA1F,CACNA1S,CACNA2D1,CACNB3,CACNB4,GRIN1,GRIN2A,GRIN2B,GRIN2C,GRIN2D |
| GO: Molecular Function | voltage‑gated channel activity | CACNA1C,CACNA1D,CACNA1F,CACNA1S,CACNA2D1,CACNB3,CACNB4,GRIN1,GRIN2A,GRIN2B,GRIN2C,GRIN2D |
| GO: Molecular Function | gated channel activity | CACNA1C,CACNA1D,CACNA1F,CACNA1S,CACNA2D1,CACNB3,CACNB4,GRIN1,GRIN2A,GRIN2B,GRIN2C,GRIN2D,PLCB4 |
| GO: Molecular Function | monoatomic cation channel activity | CACNA1C,CACNA1D,CACNA1F,CACNA1S,CACNA2D1,CACNB3,CACNB4,GRIA2,GRIN1,GRIN2A,GRIN2B,GRIN2C,GRIN2D,PLCB4,CACNG3,CACNG2,CACNG4,CACNG8,CACNG7,CACNG6,CACNA2D4,GRIN3A,GRIN3B |
| GO: Molecular Function | monoatomic ion channel activity | CACNA1C,CACNA1D,CACNA1F,CACNA1S,CACNA2D1,CACNB3,CACNB4,GRIN1,GRIN2A,GRIN2B,GRIN2C,GRIN2D,PLCB4,CACNG3,CACNG2,CACNG4,CACNG8,CACNG7,CACNG6,CACNA2D4,GRIN3A,GRIN3B |
| GO: Molecular Function | channel activity | CACNA1C,CACNA1D,CACNA1F,CACNA1S,CACNA2D1,CACNB3,CACNB4,GRIN1,GRIN2A,GRIN2B,GRIN2C,GRIN2D,PLCB4,CACNG3,CACNG2,CACNG4,CACNG8,CACNG7,CACNG6,CACNA2D4,GRIN3A,GRIN3B |
| GO: Molecular Function | passive transmembrane transporter activity | GRIA1,GRIA2,GRIA3,GRIA4,GRID1,GRIN1,GRIN2A,GRIN2B,GRIN2C,GRIN2D,NOS1,GRIN3A,GRIN3B |

| **Table S6.** The details of the 64 hub genes in the interconnected PPI network. | |
| --- | --- |
| **Hub genes** | |
| **Gene symbol** | **Betweenness** |
| CACNG8 | 59283 |
| CACNB4 | 785 |
| GRIN1 | 2902 |
| DLG4 | 1742 |
| GRIN3A | 116443 |
| PRKACG | 5568 |
| CACNA1F | 778 |
| GRIA1 | 2890 |
| GRIN2D | 2906 |
| CACNG3 | 10368 |
| CACNA1S | 779 |
| GRIN2A | 2903 |
| GRIA2 | 2891 |
| DLG3 | 1741 |
| CACNG2 | 10369 |
| PPP1CC | 5501 |
| CACNG6 | 59285 |
| CACNA1D | 776 |
| CAMK2B | 816 |
| GRIN3B | 116444 |
| DLG1 | 1739 |
| CACNG7 | 59284 |
| CACNA2D4 | 93589 |
| CNIH3 | 149111 |
| GRIA3 | 2892 |
| CACNB3 | 784 |
| GRIA4 | 2893 |
| GRIN2C | 2905 |
| PRKACB | 5567 |
| CACNA2D1 | 781 |
| CACNG4 | 27092 |
| CNIH2 | 254263 |
| PPP1CA | 5499 |
| CACNA1C | 775 |
| GRIN2B | 2904 |
| HSPA2 | 3306 |
| PRKACA | 5566 |
| SHANK3 | 85358 |
| DLGAP1 | 9229 |
| SHANK1 | 50944 |
| SHANK2 | 22941 |
| INPP5A | 3632 |
| MINPP1 | 9562 |
| IPMK | 253430 |
| ITPKA | 3706 |
| KIF5B | 3799 |
| KIF5C | 3800 |
| KIF5A | 3798 |
